# Genome-wide analysis of mono-, di- and trimethylation of histone H3 lysine 4 in *Arabidopsis thaliana*

**DOI:** 10.1186/gb-2009-10-6-r62

**Published:** 2009-06-09

**Authors:** Xiaoyu Zhang, Yana V Bernatavichute, Shawn Cokus, Matteo Pellegrini, Steven E Jacobsen

**Affiliations:** 1 Department of Plant Biology, University of Georgia, Green Street, Athens, GA 30602, USA; 2 Department of Molecular, Cell and Developmental Biology, University of California, Los Angeles, Charles E Young Drive South, Los Angeles, CA 90095, USA; 3 Molecular Biology Institute, University of California, Los Angeles, Charles E Young Drive South, Los Angeles, CA 90095, USA; 4 Howard Hughes Medical Institute, University of California, Los Angeles, Charles E Young Drive South, Los Angeles, CA 90095, USA

## Abstract

**Background:**

Post-translational modifications of histones play important roles in maintaining normal transcription patterns by directly or indirectly affecting the structural properties of the chromatin. In plants, methylation of histone H3 lysine 4 (H3K4me) is associated with genes and required for normal plant development.

**Results:**

We have characterized the genome-wide distribution patterns of mono-, di- and trimethylation of H3K4 (H3K4me1, H3K4me2 and H3K4me3, respectively) in *Arabidopsis thaliana *seedlings using chromatin immunoprecipitation and high-resolution whole-genome tiling microarrays (ChIP-chip). All three types of H3K4me are found to be almost exclusively genic, and two-thirds of *Arabidopsis *genes contain at least one type of H3K4me. H3K4me2 and H3K4me3 accumulate predominantly in promoters and 5' genic regions, whereas H3K4me1 is distributed within transcribed regions. In addition, H3K4me3-containing genes are highly expressed with low levels of tissue specificity, but H3K4me1 or H3K4me2 may not be directly involved in transcriptional activation. Furthermore, the preferential co-localization of H3K4me3 and H3K27me3 found in mammals does not appear to occur in plants at a genome-wide level, but H3K4me2 and H3K27me3 co-localize at a higher-than-expected frequency. Finally, we found that H3K4me2/3 and DNA methylation appear to be mutually exclusive, but surprisingly, H3K4me1 is highly correlated with CG DNA methylation in the transcribed regions of genes.

**Conclusions:**

H3K4me plays widespread roles in regulating gene expression in plants. Although many aspects of the mechanisms and functions of H3K4me appear to be conserved among all three kingdoms, we observed significant differences in the relationship between H3K4me and transcription or other epigenetic pathways in plants and mammals.

## Abstract 2

Analysis of the genome-wide distribution patterns of histone H3 lysine4 methylation in Arabidopsis thaliana seedlings shows that it has widespread roles in regulating gene expression.

## Background

Post-translational modifications of histones play important roles in maintaining normal transcription patterns by directly or indirectly affecting the structural properties of the chromatin. Histone modifications are highly complex due to the large number of residues that can be modified as well as the variety of modification types (for example, methylation, acetylation, phosphorylation and ubiquitination, and so on) [1]. In addition, in the case of lysine methylation, a lysine residue can be mono-, di- or trimethylated with potentially different effects on chromatin structure [2-4]. Some histone modifications can directly alter chromatin structure. For example, acetylation of specific residues in the globular core domains of histones weakens the histone-DNA interactions, resulting in a relatively 'open' chromatin structure that facilitates transcription [5,6]. In contrast, other modifications (such as lysine methylation on the amino-terminal tail of H3) do not grossly affect chromatin structure *per se*, but interact with additional factors. For example, several groups of proteins have been shown to preferentially bind histone H3 methylated at lysine 4 (H3K4me): the human chromatin remodeling and assembly factor hCHD1 (human homolog of Chromodomain helicase DNA binding protein 1) binds H3K4me through its chromodomain [7,8], the chromatin remodeling complex NURF (Nucleosome remodeling factor) binds H3K4me through the PHD (plant homeodomain) domain of its large subunit BPTF (Bromodomain PHD finger transcription factor) [9], the H3K9me3 and H3K36me3 demethylase JMJD2A (Jumonji domain containing 2A) binds H3K4me (and H4K20me3) through its Tudor domain [10,11], and members of the ING (Inhibitor of growth) family of tumor suppressor proteins bind H3K4me through the PHD domain [12,13].

Four lysine residues on histone H3 were found to be methylated in *Arabidopsis thaliana *by mass spectrometry studies (H3K4, H3K9, H3K27 and H3K36) [14,15]. Di-methylation of histone H3 lysine 9 (H3K9me2) is required for the transcriptional silencing of transposons and other repetitive sequences [16,17], whereas H3K27me3 is primarily involved in the developmental repression of endogenous genes [18-21]. Recent genome-wide profiling studies in *Arabidopsis *have shown that H3K9me2 is highly enriched in the pericentromeric heterochromatin where transposons and other repeats cluster [22-25], whereas H3K27me3 is mostly distributed in the transcribed regions of a large number of euchromatic genes and bound by the chromodomain-containing protein LIKE HETEROCHROMATIN PROTEIN-1 (LHP1) [23,26,27]. H3K36me is required for normal plant development, but the genome-wide distribution of this modification and its role in transcriptional regulation remain unclear [28-31]. Finally, H3K4me2 is primarily distributed in endogenous genes but not transcriptionally silent transposons, as shown by a previous study of a 1-Mb heterochromatic region in *Arabidopsis *[22].

Only one H3K4 methyltransferase (SET1; SET domain containing 1) has been identified in yeast (*Saccharomyces cerevisiae*), and it has been proposed the differential methylation of H3K4 can be attributed to the kinetics of the dissociation of SET1 from the elongating RNA polymerase [32]. Multiple putative H3K4 methyltransferases homologous to SET1 have been identified in *Arabidopsis *[33-36]. Several lines of evidence suggest that in *Arabidopsis *distinct H3K4 methyltransferase complexes may also contribute to the differential accumulation of H3K4me1, H3K4me2 and H3K4me3 at specific loci. For example, loss of the H3K4 methyltransferase ATX1 (*Arabidopsis *homolog of Trithorax 1) leads to a mild reduction in global H3K4me3 level and eliminates H3K4me3 at specific loci, but has no detectable effect on H3K4me2 [37]. In contrast, the loss of a closely related H3K4 methyltransferase, ATX2, results in locus-specific defects in H3K4me2 but does not appear to affect H3K4me3 [38]. Examination of H3K4me levels at several genes revealed that the types of H3K4me present at individual genes may differ significantly [38,39]. Interestingly, the *atx1 *mutant exhibits several developmental abnormalities, whereas the *atx2 *mutant is phenotypically normal [38-40]. Furthermore, results from transcriptional profiling studies indicated that ATX1 and ATX2 likely regulate two largely non-overlapping sets of genes [38]. It therefore appears that there may be significant differences in the mechanism, localization and function H3K4me1, H3K4me2 and H3K4me3.

Here we report a genome-wide analysis of H3K4me1, H3K4me2 and H3K4me3 in *Arabidopsis *using chromatin immunoprecipitation (ChIP) and whole-genome tiling microarrays (ChIP-chip). We found that all three types of H3K4me are distributed exclusively within genes and their promoters, and that approximately two-thirds of genes contain at least one type of H3K4me. In addition, H3K4me3, H3K4me2 and H3K4me1 are distributed with a 5'-to-3' gradient along genes, where H3K4me3 and H3K4me2 are enriched in the promoters and 5' end of transcribed regions with H3K4me3 distributed slightly upstream of H3K4me2, and H3K4me1 is depleted in promoters but enriched in the transcribed regions with an apparent 3' bias. Interestingly, we found that genes associated with different combinations of H3K4me are expressed at different levels and with different degrees of tissue specificity. Furthermore, genome-wide comparisons between H3K4me and other epigenetic marks revealed preferential co-localization between H3K4me2 and H3K27me3, and between H3K4me1 and CG DNA methylation in the transcribed regions of genes. Finally, the relationship between H3K4me and DNA methylation was further examined by genome-wide profiling of H3K4me in a DNA methylation mutant. The results suggested that H3K4me and DNA methylation may not directly interfere with each other in *Arabidopsis*, and that these two epigenetic pathways interact primarily through transcription.

## Results and discussion

### Genome-wide profiling of H3K4me1, H3K4me2 and H3K4me3

*Arabidopsis *chromatin enriched for H3K4me was isolated by ChIP using antibodies that specifically recognize H3K4me1, H3K4me2 and H3K4me3 (Figure S1 in Additional data file 1). As a control, nucleosomal DNA was isolated by ChIP using an antibody against histone H3 regardless of its modifications. H3K4me ChIP samples were compared to the control nucleosomal DNA by hybridization to Affymetrix whole-genome tiling microarrays that represent approximately 97% of the sequenced *Arabidopsis *genome at 35-bp resolution.

H3K4me1, H3K4me2 and H3K4me3 regions identified here are highly consistent with results from recently published studies [38] (Figure S2 in Additional data file 1). In addition, real-time PCR validations were performed at a number of randomly chosen loci, all of which yielded results consistent with the ChIP-chip data (Figure S3 in Additional data file 1). Finally, only 0.10%, 0.66% and 0.57% of the chloroplast genome was falsely identified as containing H3K4me1, H3K4me2 and H3K4me3, respectively. Taken together, these results indicate that the ChIP-chip data here provide an accurate representation of the genome-wide distribution of H3K4me with a relatively low false positive rate.

### H3K4me1, H3K4me2 and H3K4me3 accumulate exclusively in genes

**Figure 1:**
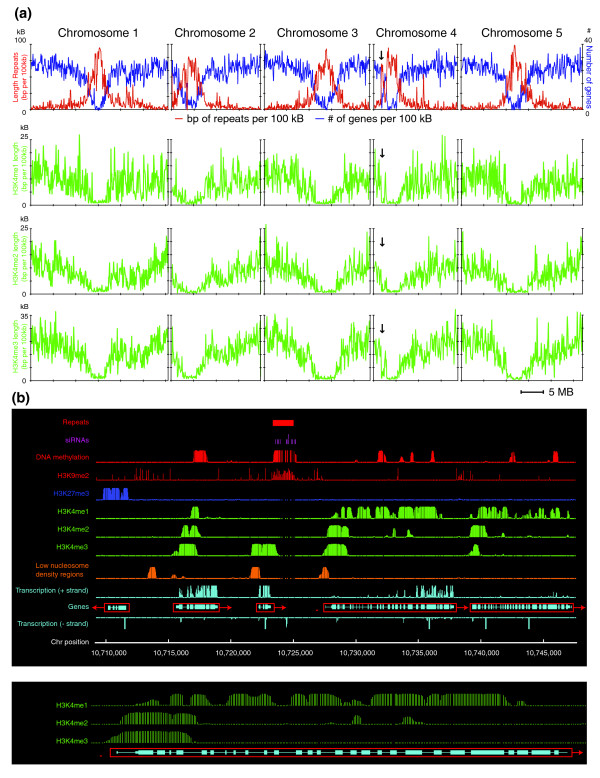
Distribution of H3K4me in the *Arabidopsis *genome. **(a) **Chromosomal distribution of H3K4me. Top row: the total length of repetitive sequences (y-axis, left-side scale) and number of genes per 100 kb (y-axis, right-side scale). Bottom panels: chromosomal distribution of H3K4me1, H3K4me2 and H3K4me3. X-axis: chromosomal position; y-axis: the total length of genomic regions containing H3K4me1, H3K4me2 and H3K4me3 per 100 kb, respectively. Arrows indicate the heterochromatic knob on chromosome 4. **(b) **Local distribution of H3K4me1, H3K4me2, H3K4me3, other epigenetic marks (DNA methylation, H3K9me2, H3K27me3, nucleosome density, small RNAs) and transcription activity in an approximately 40-kb euchromatic region on chromosome 1. Repetitive sequences are shown as filled red boxes on top. Individual genes are shown in open red boxes (arrows indicate direction of transcription; filled light blue boxes, exons; light blue lines, introns). Distribution of H3K4me on the gene labeled by a red asterisk is enlarged and shown in detail at the bottom.

A total of 15,475 (7.77 Mb) H3K4me1, 12,781 (7.17 Mb) H3K4me2 and 15,894 (14.48 Mb) H3K4me3 regions were identified as described above, representing 6.45%, 6.0% and 12.1% of the sequenced nuclear genome, respectively. All three types of H3K4me are highly enriched in the gene-rich euchromatin and absent from pericentromeric heterochromatin regions where transposons and other repetitive sequences cluster (Figure 1a). Such a euchromatic distribution may largely reflect the fact that H3K4me1, H3K4me2 and H3K4me3 localize almost exclusively in genes: 96.7%, 93.3% and 95.7% of all H3K4me1, H3K4me2 and H3K4me3 regions, respectively, are in or overlap with transcribed regions of genes or their promoters (defined as the 200-bp regions upstream of transcription start sites). Only a small fraction of the remaining H3K4me1, H3K4me2 and H3K4me3 regions (0.6%, 1.3% and 1.5% of total, respectively) overlap with intergenic repetitive sequences such as transposons. The distribution of HK4me in a representative eukaryotic region is shown in Figure 1b.

### Differential distribution of H3K4me1, H3K4me2 and H3K4me3 within genes

A total of 18,233 genes (approximately 68.0% of all annotated genes) were found to contain H3K4me in their promoters and/or transcribed regions, including 8,571 with H3K4me1, 10,396 with H3K4me2 and 14,712 with H3K4me3. The distribution patterns of H3K4me at the 5' regions of genes were determined by aligning genes by their transcription start sites, and the percentage of genes containing H3K4me in their promoters and the 5' transcribed regions was determined. Similarly, the distribution patterns of H3K4me at the 3' regions of genes were determined by aligning genes by the 3' end of their transcribed regions. These analyses were performed on a set of 5,809 genes that meet the following two criteria. First, they are located 1 kb or more away from the upstream and downstream genes such that ambiguity introduced by neighboring genes can be minimized. Second, they are longer than 1 kb so that there is sufficient gene space to determine the distribution of H3K4me. We further classified the 5,809 genes into four groups according to their length: long genes (>4 kb, 691 genes), intermediate genes (3 to 4 kb, 828 genes; 2 to 3 kb, 1,768 genes) and short genes (1 to 2 kb, 2,522 genes).

**Figure 2:**
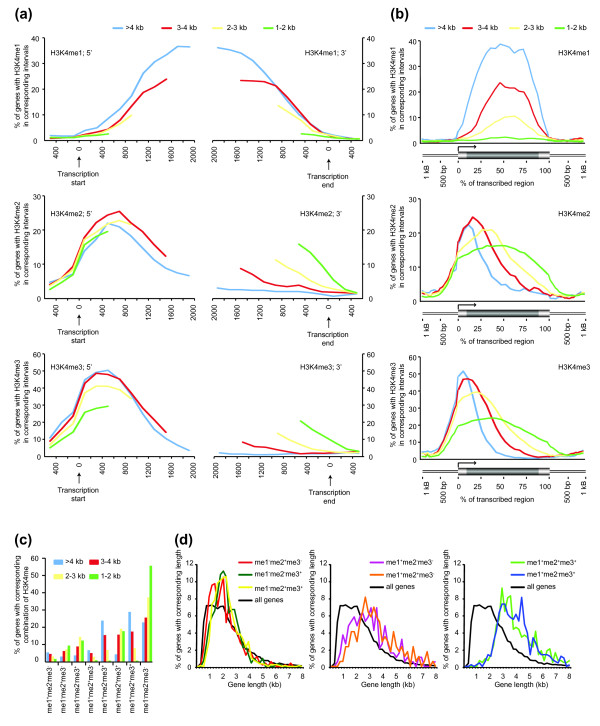
Distribution of H3K4me relative to genes. **(a) **Distribution of H3K4me at the 5' and 3' ends of genes. 'Isolated' genes are divided into four groups according to their length (see text for details). Genes belonging to each length group were aligned at the transcription start sites, and the percentage of genes containing H3K4me in their promoters or 5' ends is determined at 200-bp intervals (left y-axis). Similarly, genes belonging to each length group were aligned at the end of transcribed regions, and the percentage of genes containing H3K4me in their 3' ends or downstream flanking regions is determined at 200-bp intervals (right y-axis). The first and last 500 bp, 1 kb, 1.5 kb and 2 kb are shown for genes that are 1 to 2 kb, 2 to 3 kb, 3 to 4 kb and >4 kb in length, respectively. **(b) **Distribution of H3K4me across genes. Each gene (thick horizontal bar) was divided into 20 intervals (5% each interval), and the 1-kb regions upstream and downstream of each gene (thin horizontal bars) were divided into 50-bp intervals. The percentage of genes with H3K27me3 in each interval was graphed (y-axis). **(c) **Relationship between gene length and H3K4me. Genes are divided into eight categories according to the combination of H3K4me (see text for details), and the percentage of genes within each length group that are associated with a particular combination of H3K4me is shown (y-axis). **(d) **Length distribution of genes associated with different combinations of H3K4me. X-axis: gene length in kb (200 bp per bin); y-axis: the percentage of genes associated with a particular combination of H3K4me that are of the corresponding length. A small number of genes longer than 8 kb are not shown.

The distribution patterns of H3K4me on long genes are shown in Figure 2a. H3K4me1 is present at relatively low level at the 5' and 3' termini of transcribed regions, but is enriched in the internal regions with a slight 3' bias. In contrast, H3K4me2 and H3K4me3 are both enriched in the 5' end with H3K4me3 distributed slightly upstream of H3K4me2. Both H3K4me2 and H3K4me3 are also enriched in the promoters (200 bp upstream of transcription start sites) and 5' flanking regions (200 to approximately 400 bp upstream of transcription start sites), but are absent in the 3' half of the transcribed regions or the 3' flanking regions of the long genes.

A comparison of the distribution patterns of H3K4me on long genes and intermediate or short genes revealed several common features as well as some interesting differences. First, as gene length decreases, significantly smaller fractions of genes were found to contain H3K4me1, but the relative position of H3K4me1 in genes (that is, internal regions with a 3' bias) remains similar. Second, the distribution patterns of both H3K4me2 and H3K4me3 at the 5' ends of short or intermediate genes are largely similar to those on long genes, although the shortest genes seem to contain a lower level of H3K4me3 at the 5' end. Third, as gene length decreases, significantly more genes were found to contain H3K4me2 and H3K4me3 in their 3' regions. For example, in the last 200 bp, 10.8- and 13.3-fold more short genes contain H3K4me2 and H3K4me3 than long genes, respectively.

In order to obtain a more continuous view of the distribution of H3K4me, we analyzed the average distribution levels of H3K4me across entire genes. To do this, we divided the transcribed region of each gene into 20 bins (5% of the gene length per bin), and divided the 1-kb upstream and downstream flanking regions of each gene into 20 bins (50 bp per bin). The percentage of genes containing H3K4me in each bin was then determined (Figure 2b). Consistent with the results described above, H3K4me1 is highly enriched within the transcribed regions, but it is present at very low levels in promoters and 3' flanking regions. In addition, H3K4me1 is present at significantly higher levels and spans broader regions on longer genes. In contrast, H3K4me2 and H3K4me3 are enriched in promoters and the 5' half of transcribed regions, at comparable levels on genes with different lengths. Although H3K4me2 and H3K4me3 extend further towards the 3' end on shorter genes relative to gene length, the absolute positions remain virtually constant: regardless of gene length, the highest levels of H3K4me2 and H3K4me3 were found at approximately 600 to 800 bp and 400 to 600 bp downstream of transcription start sites, respectively (Figure 2a). In addition, for genes in all the length groups, H3K4me2 and H3K4me3 appear to be enriched (that is, present at the same or higher levels as they are at transcription start sites) downstream of transcription start sites for approximately 1.5 kb and 1 kb, respectively (Figure 2a).

The observation that H3K4me2 and H3K4me3 appear to cover the 5' regions of genes for a relatively constant length suggests that the length of a given gene may affect the association of this gene with different types of H3K4me, in particular H3K4me1. For example, while all three types of H3K4me are positively correlated with gene length (Figure 2b), such a relationship is significantly more pronounced for H3K4me1. To further study the relationship between gene length and H3K4me, we classified the 5,809 genes into 8 categories based on the 8 possible combinations of their associated H3K4me: H3K4me1 only (me1^+^me2^-^me3^-^), H3K4me2 only (me1^-^me2^+^me3^-^), H3K4me3 only (me1^-^me2^-^me3^+^), H3K4me1 and H3K4me2 but no H3K4me3 (me1^+^me2^+^me3^-^), H3K4me1 and H3K4me3 but not H3K4me2 (me1^+^me2^-^me3^+^), H3K4me2 and H3K4me3 but not H3K4me2 (me1^-^me2^+^me3^+^), H3K4me1, H3K4me2 and H3K4me3 (me1^+^me2^+^me3^+^), and no H3K4me (me1^-^me2^-^me3^-^). The frequencies of occurrences of these combinations within each length group were then determined. As shown in Figure 2c, all combinations that include H3K4me1 (regardless of H3K4me2 and H3K4me3) showed a strong positive correlation with gene length, and all combinations of H3K4me2 and H3K4me3 (in the absence of H3K4me1) showed a negative correlation with gene length. In addition, genes associated with H3K4me1 (me1^+^me2^-^me3^-^, me1^+^me2^+^me3^-^, me1^+^me2^-^me3^+^, me1^+^me2^+^me3^+^) are generally longer than average, with me1^+^me2^-^me3^+ ^and me1^+^me2^+^me3^+ ^genes being significantly longer and including very few genes shorter than 2 kb (Figure 2d). In summary, by every measure, longer genes show higher levels of H3K4me1.

The distribution patterns of H3K4me2 and H3K4me3 described here are similar to results from analyzing genes on chromosomes 4 and 10 in rice [41]. That is, in both species, H3K4me2 and H3K4me3 are enriched in the promoters and the 5' ends of transcribed regions, with H3K4me3 peaking slightly upstream of H3K4me2 (at approximately 400 to 600 bp and approximately 600 to 800 bp downstream of transcription start sites, respectively; Figure 2a). These results suggest that H3K4me2 and H3K4me3 may be involved in both transcription initiation and the early stages of transcription elongation. In contrast, the internal distribution of H3K4me1 observed here suggests that H3K4me1 might be primarily involved in the elongation step during the transcription of longer genes. Alternatively, the apparent preferential accumulation of H3K4me1 in the transcribed regions may be because this modification is reduced at gene ends (that is, H3K4 is preferentially di- or trimethylated at the 5' ends and unmethylated at the 3' ends).

### Association of different combinations of H3K4me1, H3K4me2 and H3K4me3 with differential gene expression patterns

**Figure 3:**
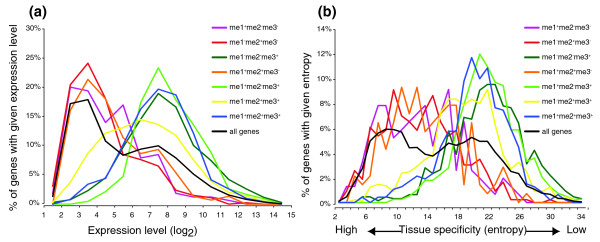
Genes with different expression levels and patterns are associated with different combinations of H3K4me. **(a) **Distribution of expression levels of genes associated with different combinations of H3K4me. X-axis: gene expression level determined in a previous study (log_2 _scale) [42]. Y-axis: the percentage of genes with corresponding H3K4me combination and expression level. **(b) **The degree of tissue-specific expression of genes associated with different combinations of H3K4me, as measured by entropy (x-axis). Y-axis: the percentage of genes with corresponding H3K4me combination and entropy values.

To further test the relationship between H3K4me and transcription, we compared the expression level and tissue specificity of genes associated with different combinations of H3K4me, using a previously published expression profiling dataset [42]. Of the 5,809 genes described above, 5,479 were analyzed here, as expression data were available for these genes. As shown in Figure 3a, me1^+^me2^-^me3^+^, me1^+^me2^+^me3^+ ^and me1^-^me2^-^me3^+ ^genes are highly expressed, whereas me1^+^me2^-^me3^-^, me1^-^me2^+^me3^- ^and me1^+^me2^+^me3^- ^genes are expressed at very low levels. The me1^-^me2^+^me3^+ ^group includes genes with a wide range of expression levels and seems to be enriched for moderately expressed genes. In addition, me1^+^me2^-^me3^+^, me1^+^me2^+^me3^+ ^and me1^-^me2^-^me3^+ ^genes exhibit very low levels of tissue specificity, while me1^+^me2^-^me3^-^, me1^-^me2^+^me3^- ^and me1^+^me2^+^me3^- ^genes are highly tissue specific (Figure 3b). Taken together, these results suggest that H3K4me3 is associated with and likely plays important roles in active transcription. H3K4me1 and H3K4me2, in the absence of H3K4me3, are preferentially associated with tissue-specific genes that are generally not expressed at the developmental stage assayed in this study. These results are consistent with previous reports that although H3K4me2 is generally associated with genes in *Arabidopsis*, its presence does not always correlate with active transcription [37].

### Relationship between H3K4me and H3K27me3

In *Drosophila*, the Trithorax (TRX) family of H3K4 methyltransferases and the Enhancer of Zeste (E(z)) family of H3K27 methyltransferases function antagonistically to activate or repress a largely overlapping set of genes, respectively [43,44]. Interestingly, many genes are associated with both H3K4me and H3K27me3 in mammalian stem cells, and such a 'bivalent' histone modification has been suggested to play an important role in stem cell renewal and differentiation [45]. Similarly, the co-existence and antagonistic functions of H3K4me3 and H3K27me3 have been described at the *FLC *and *AGAMOUS *genes in *Arabidopsis *[38,39,46-48]. We have indeed detected H3K4me2, H3K4me3 and H3K27me3 at the *FLC *gene. However, we found that *AGAMOUS *contains a low level of H3K4me2 but no significant level of H3K4me3. This apparent discrepancy is likely due to the different tissues used in the experiments: young seedlings were used in this studywhereas a previous study used mature rosettes (Z Avramova, personal communication).

We have previously found that H3K27me3 is associated with 4,000 to 5,000 tissue-specific genes in their repressed state in *Arabidopsis *[26]. In order to test whether a preferential association of H3K4me with H3K27me3 exists that could indicate a functional connection, we first determined the fraction of genes with each combination of H3K4me that are also associated with H3K27me3. As shown in Table 1, we found that me1^-^me2^-^me3^- ^and me1^-^me2^+^me3^- ^genes are associated with H3K27me3 more frequently than expected. In addition, the association frequencies of me1^+^me2^-^me3^-^, me1^+^me2^+^me3^- ^and me1^-^me2^+^me3^+ ^genes with H3K27me3 are all lower than expected. Finally, me1^-^me2^-^me3^+^, me1^+^me2^-^me3^+^, and me1^+^me2^+^me3^+ ^genes are even more depleted of H3K27me3. It should be noted that the differences in transcription levels cannot fully account for the differential association of H3K4me genes with H3K27me3. For example, the me1^-^me2^-^me3^- ^and me1^-^me2^+^me3^- ^genes are significantly more frequently associated with H3K27me3 than me1^+^me2^-^me3^- ^and me1^+^me2^+^me3^- ^genes, but these four categories of genes are expressed at very similar levels (Figure 3). The relationship between H3K4me and H3K27me3 was further examined by directly testing whether they co-localize to the same genomic regions. To do this, we determined the presence of each type of H3K4me in H3K27me3-containing genomic regions. As a control, we also determined the presence of H3K4me in a set of randomly chosen regions with the same length and genomic distributions of H3K27me3-containing regions. As shown in Table 2, whereas H3K4me1 and H3K4me3 are significantly depleted in H3K27me3-containing regions, H3K4me2 was found to overlap with H3K27me3 slightly more frequently than with random control regions.

**Table 1:** Co-localization of H3K4me and H3K27me3 in genes

	Total	H3K27me3 target genes	Observed	Enriched for H3K27me3 target genes?*	Depleted of H3K27me3 target genes?*
me1^+^me2^-^me3^-^	179	31	17.32%	No (*P *= 1)	Yes (*P *= 1.6 × 10^-5^)
me1^-^me2^+^me3^-^	445	206	46.29%	Yes (*P *< 10^-10^)	No (*P *= 1)
me1^-^me2^-^me3^+^	675	69	10.22%	No (*P *= 1)	Yes (*P *< 10^-10^)
me1^-^me2^+^me3^-^	171	33	19.30%	No (*P *= 1)	Yes (*P *= 3.0 × 10^-4^)
me1^+^me2^-^me3^+^	437	14	3.20%	No (*P *= 1)	Yes (*P *< 10^-10^)
me1^-^me2^+^me3^+^	954	173	18.13%	No (*P *= 1)	Yes (*P *< 10^-10^)
me1^+^me2^+^me3^+^	507	16	3.16%	No (*P *= 1)	Yes (*P *< 10^-10^)
me1^-^me2^-^me3^-^	2,441	1,266	51.86%	Yes (*P *< 10^-10^)	No (*P *= 1)

*Of the 5,809 genes, 1,808 (31.12%) contain H3K27me3. If the localizations of H3K4me and H3K27me3 are independent of each other, roughly 31.12% of the genes with each H3K4me combination should also contain H3K27me3.

**Table 2:** Co-localization of H3K4me and H3K27me3 in the same genome regions

	Total regions	Overlap with H3K27me3	%	Random overlapping*	Enriched for H3K27me3?	Depleted of H3K27me3?
H3K4me1	15,475	178	1.15	4.78%	No (*P *= 1)	Yes (*P *< 10^-10^)
H3K4me2	12,781	881	6.89	6.09%	Yes (*P *= 1.0 × 10^-4^)	No (*P *= 1)
H3K4me3	15,894	572	3.60	7.12%	No (*P *= 1)	Yes (*P *< 10^-10^)

*For each H3K27me3-containing genomic region, a genomic region of the same length was randomly selected within its 10-kb upstream or downstream flanking regions. The set of random control regions thus resemble the H3K27me3 in both length and chromosomal distributions. The overlapping frequencies of the random control regions with H3K4me-containing regions were then determined.

It should be noted that the starting materials in this study (young seedlings) included many distinct cell types. It is likely that some genes are associated with H3K4me3 when they are expressed in some cell types, but are associated with H3K27m3 elsewhere when they are transcriptionally repressed. It is therefore possible that the low frequency of co-localization between H3K4me3 and H3K27me3 described here may still represent an overestimate. It is also possible, however, that co-localization of H3K4me3 and H3K27me3 at a given gene only occurs in specific cell types or during certain developmental stages. If this is the case, our results generated using mixed cell types from a single development stage could represent a gross underestimate of the prevalence of bivalent chromatin modification in plants. Future studies at cell-specific levels should more directly address the exact extent to which plant genes are bivalently modified. In any event, our results seem to indicate a mutually exclusive relationship between H3K4me3 and H3K27me3 at many genes in *Arabidopsis *seedlings. In animals, the H3K4 demethylase JARID1A (Jumonji, AT rich interactive domain 1A)/RBP2 (Retinol binding protein 2) is recruited to genomic targets through its interaction with the H3K27me3 methyltransferase complex Polycomb repressive complex (PRC) 2, where RBP2 mediates transcriptional repression by demethylating H3K4me3 to H3K4me2 (and to a lesser extent, H3K4me2 to H3K4me1) [49,50]. In addition, the H3K4me3-specific demethylase JARID1D interacts with Ring6a (Really interesting new gene 6a)/MBLR (Mel18 and Bmi1-like RING finger protein), which is closely related to the PRC1 components Bmi1 (B Lymphoma Mo-MLV insertion region 1) and Mel18 [51]. Interestingly, two *Arabidopsis *RING finger proteins, AtRING1a and AtRING1b, have been recently found to interact with the H3K27me3 methyltransferase CURLY LEAF and the H3K27me3-binding protein LIKE HETEROCHROMATIN PROTEIN1, and are required for the transcriptional repression of H3K27me3 target genes [52]. The general mutual exclusion between H3K4me3 and H3K27me3 as well as the more frequent overlap of H3K4me2 and H3K27me3 suggest that similar mechanisms might also function in plants. That is, plant H3K4me3 demethylase(s) may function in transcriptional repression by interacting with PRC1 and/or PCR2. If this is the case, a fraction of the H3K4me2 in the *Arabidopsis *genome could be the demethylation product of H3K4me3.

We also observed that H3K4me1 tended not to co-localize with H3K27me3. One contributing factor could be the differential distribution patterns of these histone modifications along genes: H3K4me1 tends to be present at the 3' half of long genes, whereas H3K27me3 does not exhibit similar preferences for either location within genes or gene length (Figure S4 in Additional data file 1). Furthermore, H3K4me1 was present more frequently on ubiquitously expressed housekeeping genes, while H3K27m3 was more frequently present on tissue-specific genes.

### Relationship between H3K4me and DNA methylation

Cytosine DNA methylation is an epigenetic silencing mechanism important for the developmental regulation of endogenous genes and the transcriptional silencing of transposons [53-56]. A mechanistic relationship between DNA methylation and H3K4me has been described in mammals, where the DNA methyltransferase (DNMT) homolog DNMT3L specifically interacts with histone H3 containing unmethylated lysine 4 [57]. That DNMT3L also binds and stimulates the activity of the *de novo *DNA methyltransferase DNMT3A suggests that H3 with unmethylated K4 may play a role in targeting *de novo *DNA methylation in mammals [57-59]. However, a distinct small interfering RNA (siRNA)-directed pathway is responsible for *de novo *DNA methylation in plants [60-62], and an interaction between DNA methyltransferase and histone has not been reported.

Three DNA methylation pathways have been described in plants: METHYLTRANSFERASE 1 (MET1) is a homolog of mammalian DNMT1 and primarily functions in maintaining DNA methylation in the CG sequence context ('CG methylation') [63-66]. The DOMAIN REARRANGED METHYLASE (DRM) (homologous to mammalian DNMT3) interacts with the siRNA pathway and is required for *de novo *DNA methylation in all sequence contexts as well as the maintenance of DNA methylation in the CHH context (H = A, C or T; 'CHH methylation') [60-62]. The CHROMOMETHYLASE3 is specific to plant genomes and interacts with the H3K9me2 pathway to maintain DNA methylation in the CHG sequence context ('CHG methylation') [67,68].

The genome-wide distribution of DNA methylation in *Arabidopsis *has been determined by a number of studies using microarray analyses or ultra-high-throughput deep sequencing of bisulfite treated DNA [22,25,69-77]. Results from these studies are largely consistent: CG, CHG and CHH methylation is highly enriched in transposons and other repetitive sequences, suggesting that the RNA interference, H3K9me2 and DNA methylation pathways function together in the transcriptional repression at these loci. DNA methylation is generally depleted in the promoters and 5' ends of endogenous genes. However, over one-third of *Arabidopsis *genes contain DNA methylation exclusively in the CG sequence context that is enriched in the 3' half of their transcribed regions (termed 'body-methylation'). Most body-methylated genes are expressed at moderate to high levels, and it is therefore unclear whether CG methylation alone in the transcribed regions of genes plays a direct and significant repressive role in transcription.

In order to determine the relationship between DNA methylation and H3K4me in *Arabidopsis*, we compared DNA methylation levels in genomic regions containing H3K4me to the whole-genome average of DNA methylation. As shown in Table 3, CHG and CHH methylation is significantly depleted in genomic regions containing H3K4me1, H3K4me2 or H3K4me3. CG methylation is also significantly depleted in H3K4me2- and H3K4me3-containing regions. In stark contrast, we found that CG methylation is highly enriched in H3K4me1-containing regions (Table 3). In addition, nearly two-thirds of H3K4me1-containing regions (8,841 of 14,599, approximately 60.6%) with two or more CG dinucleotides are methylated at two or more CG sites, compared to approximately 7.0% (842 of 12,100) and approximately 11.7% (1,750 of 14,918) for H3K4me2- and H3K4me3-containing regions, respectively.

**Table 3:** The percentage of cytosine residues that are methylated in CG, CHG or CHH sequence contexts in H3K4me-containing genomic regions*

H3K4me	Chromosome	CG	CHG	CHH
H3K4me1	1	41.78%	0.41%	0.32%
	2	41.52%	0.42%	0.31%
	3	40.38%	0.45%	0.33%
	4	42.46%	0.42%	0.32%
	5	42.03%	0.40%	0.30%
H3K4me2	1	4.22%	0.39%	0.32%
	2	4.11%	0.38%	0.34%
	3	3.89%	0.39%	0.33%
	4	4.78%	0.36%	0.33%
	5	4.32%	0.38%	0.32%
H3K4me3	1	2.93%	0.40%	0.32%
	2	2.96%	0.45%	0.35%
	3	2.85%	0.39%	0.32%
	4	3.35%	0.44%	0.34%
	5	3.08%	0.43%	0.33%

*The genome-wide averages are: CG, 24.0%; CHG, 6.7%; CHH, 1.7%.

The low level of CHG and CHH methylation in H3K4me-containing regions can be explained by the virtual absence of siRNAs and H3K9me2 within actively transcribed endogenous genes. The lack of CG methylation in H3K4me2- and H3K4me3-containing regions could be due to an active mutual exclusion mechanism (for example, MET1 may be discouraged from chromatin containing H3K4m2 or H3K4me3) similar to what was recently described between DNA methylation and the deposition of the histone variant H2A.Z [78], or simply the differential localization of DNA methylation and H3K4me2/H3K4me3 relative to genes (a 5' bias for H3K4me2/H3K4me3 and a 3' bias for DNA methylation). The high level of CG methylation in H3K4me1-containing regions was unexpected. It is possible that CG methylation and H3K4me1 interact with each other and therefore co-localize at the 3' transcribed regions of genes. It is also possible that the overlap of these two epigenetic marks merely reflects their preferential localization in the similar regions of highly expressed genes. In either case, these results indicate that CG methylation *per se *and H3K4me1 do not appear to interfere with each other. Finally, genomic regions free of H3K4me frequently lack DNA methylation, suggesting that the absence of H3K4me alone is insufficient to trigger DNA methylation.

### Ectopic H3K4me in met1 is associated with transcriptional de-repression

In order to test whether direct mechanistic links exist between DNA methylation and H3K4me (that is, whether DNA methylation *per se *excludes H3K4me2/H3K4me3 and whether gene body methylation facilitates H3K4me1), we determined the genome-wide distribution of H3K4me in the *met1 *mutant by ChIP-chip. Previous studies have shown that loss of MET1 eliminates CG methylation as well as substantial fractions of CHG and CHH methylation, resulting in massive transcriptional reactivation of transposons [71,72,74,76,77].

**Figure 4:**
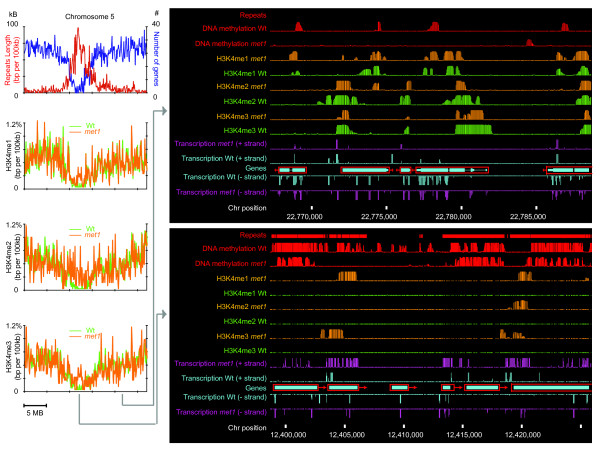
Comparisons of H3K4me accumulated in wild-type *Arabidopsis *(Wt, green) and the *met1 *mutant (light brown). Left: chromosome-level changes in H3K4me, showing the ectopic accumulation of H3K4me in the pericentromeric heterochromatin. Chromosome 5 is shown as an example (Wt, green; *met1*, light brown). X-axis: chromosome position; y-axis: the percentage of H3K4me on chromosome 5 in the corresponding region (in 100 kb bins). Right: local changes in DNA methylation, H3K4me and transcription in a euchromatic region (top right) and a heterochromatic region (bottom right) on chromosome 5. The five genes shown in the euchromatic region likely encode cellular proteins and their expression patterns are unaffected in the *met1 *mutant. These are (from left to right): At5g56210, WPP-DOMAIN INTERACTING PROTEIN 2; At5g56220, nucleoside-triphosphatase; At5g56230, prenylated rab acceptor (*PRA1*) family protein; At5g56240, unknown protein. The six genes shown in the heterochromatic region are all transposon-encoded genes. These are (from left to right): At5g32925, CACTA-like transposase; At5g32950, CACTA-like transposase, At5g32975, similar to En/Spm-like transposon protein; At5g33000, Transposable element gene; At5g33025, *gypsy*-like retrotransposon; At5g33050, *gypsy*-like retrotransposon. Note that the overexpression of At5g32950 and At5g33050 is associated with ectopic accumulation of H3K4me.

All three types of H3K4me were found to be present at much higher levels in the pericentromeric heterochromatin regions in *met1 *(Figure 4). A closer examination revealed that hyper-H3K4me in *met1 *is almost always associated with ectopic over-expression of transposons or pseudogenes (Figure 4). However, the loss of DNA methylation does not appear to directly trigger hyper-H3K4me. In contrast to the transcription-independent ectopic accumulation of H2A.Z in DNA hypomethylated regions in the *met1 *mutant [78], no major change in H3K4me was observed in genomic regions that are DNA-hypomethylated but not transcribed. This suggests that the ectopic transcriptional activity resulting from the loss of DNA methylation, but not the loss of DNA methylation *per se*, is associated with hyper-H3K4me. In addition, at nearly all genes, a complete loss of gene body methylation in *met1 *had no significant effect on H3K4me1, H3K4me2 or H3K4me3 (Figure 4), suggesting that CG methylation in genes is dispensable for the normal accumulation of H3K4me1.

## Conclusions

Our genome-wide analysis of H3K4me1, H3K4me2 and H3K4me3 led to several interesting results. First, a large number of genes were found to contain H3K4me: at a single developmental stage, approximately two-thirds of all *Arabidopsis *genes contain at least one type of H3K4me. This suggests that H3K4me may be required for the normal expression or a large number of genes in plants. Second, H3K4me1, H3K4me2 and H3K4me3 are enriched in different regions in their target genes. H3K4me2 and H3K4me3 are distributed in the promoters and 5' regions with H3K4me3 slightly more upstream, whereas H3K4me1 is mostly located within the transcribed regions. Our H3K4me3 results are highly consistent with those recently published by van Nocker and colleagues [47]. Importantly, very similar distribution patterns of H3K4me1, H3K4me2 and H3K4me3 were also found in yeast, human and other plants (for example, H3K4me2 and H3K4me3 in rice) [32,41,79-81], which suggests that many aspects of the mechanisms and functions of H3K4me may be highly conserved during evolution. Third, we found that genes with different expression levels and tissue specificity are associated with different assortments of H3K4me1, H3K4me2 and H3K4me3, suggesting that the three types of H3K4me may have different effects on chromatin structure and transcription. In particular, whereas H3K4me3 appears to be generally associated with actively transcribed genes, our results do not support a direct role of H3K4me1 and H3K4me2 in transcriptional activation: H3K4me1 and H3K4me2 do not appear to have an additive effect on H3K4me3 with regard to transcription levels and, in the absence of H3K4me3, they are not preferentially associated with active transcription. Interestingly, our observation that H3K4me2 (but not H3K4me1 or H3K4me3) often overlaps with H3K27me3 raises the possibility that the accumulation of H3K4me2 at some loci in the *Arabidopsis *genome might result from demethylation of H3K4me3 by histone demethylases associated with PcG complexes. Fourth, unlike in mammalian stem cells, H3K4me3 and H3K27me3 do not appear to preferentially co-localize on a genome-wide level in *Arabidopsis*. A second significant difference between plants and mammals is that, in mammals, H3K4me3 is present at active promoters as well as a large number of 'poised' promoters [82], whereas in plants, the presence of H3K4me3 is usually correlated with active transcription. Finally, we observed strong negative correlations between H3K4me2/H3K4me3 and all three types of DNA methylation, and between H3K4me1 and CHG and CHH DNA methylation. However, the loss of DNA methylation does not generally trigger hyper-H3K4me in the corresponding genomic region, indicating that DNA methylation *per se *may not inhibit H3K4me. Our results do suggest that DNA methylation may interfere with H3K4me indirectly through transcriptional repression, as ectopic transcription was observed in the vast majority of the cases where DNA hypomethylation and hyper-H3K4me occur at the same genes. Interestingly, H3K4me1 is highly correlated with the CG methylation that exists within the transcribed regions of genes. Although the retention of H3K4m1 in the *met1 *mutant indicates that CG DNA methylation is not required for the accumulation of H3K4me1, it is possible that H3K4me1 might play a role in the colonization of CG DNA methylation within the transcribed regions of genes.

## Materials and methods

*Arabidopsis thaliana *plants (accession Col) were grown on soil under continuous light for 3 weeks, and the aerial part of the seedlings was harvested. The *met1-3 *mutant plants were grown under the same conditions and harvested at a similar developmental stage. Chromatin was fragmented to 300 to 1,200 bp (mostly 600 to 800 bp) by sonication, and ChIP was performed as previously described using antibodies purchased from Abcam (anti-H3K4me1, ab8895; anti-H3K4me2, ab7766; anti-H3K4me3, ab8580; anti-H3, ab1791) (Cambridge, MA, USA) [26]. The specificities of anti-H3K4me antibodies were validated by dot blot analysis (Figure S1 in Additional data file 1). ChIP samples were amplified, labeled, and hybridized to microarrays as previously described [26,72]. Four biological replicates were performed for H3K4me1 and H3K4me3, and eight biological replicates were performed for H3K4me2. For each H3K4me ChIP, an H3 ChIP was performed to isolate nucleosomal control DNA. Microarray hybridization intensities from probes that match a unique genomic region were analyzed using Tilemap with the Hidden Markov model option, as previously described [83]. All raw microarray data (CEL files) have been deposited in Gene Expression Omnibus [GEO:GSE13613]. Processed data showing the enrichment of H3K4me can be viewed online [84]. The gene expression data used here were from a previous comprehensive transcriptional profiling study (data for 7- to 14-day-old seedlings were used here for analysis of gene expression levels, and data for all tissue types and developmental stages were used here to analyze tissue specificity) [42]. The gene annotations used here are according to TAIR7. Real-time PCR validation of ChIP-chip results was performed using the SYBR Green I Master kit (Roche; Indianapolis, IN, USA) on a Roche Light Cycler 480. The PCR parameters are: 1 cycle of 10 minutes at 95°C, 40 cycles of 10 s at 95°C, 10 s at 60°C, and 20 s at 72°C. PCR primer sequences are listed in Table S1 in Additional data file 1.

## Abbreviations

ATX: *Arabidopsis *homolog of Trithorax; ChIP: chromatin immunoprecipitation; DNMT: DNA methyltransferase; H3K4me: H3 methylated at lysine 4; JARID: Jumonji, AT rich interactive domain; MET1: METHYLTRANSFERASE 1; PHD: plant homeodomain; PRC: Polycomb repressive complex; RBP: Retinol binding protein; SET1: SET domain containing 1; siRNA: small interfering RNA.

## Authors' contributions

XZ, YVB and SEJ designed the experiments. YVB and XZ performed the experiments. XZ, MP and SEJ analyzed the data. SC contributed reagents/materials/analysis tools. XZ wrote the paper.

## Additional data files

The following additional data are available with the online version of this paper: Figures S1 to S4 and Table S1 (included in Additional data file 1).

## Supplementary Material

Additional data file 1 Figure S1: dot blot analysis showing the specificity of antibodies used here. Figure S2: comparison of the H3K4me distribution patterns determined here and those reported in a recent locus-specific study [38]. Figure S3: real-time PCR validation of H3K4me ChIP-chip results. Figure S4: length distribution of H3K27me3 target genes. Table S1: real-time PCR primer sequences. Click here for file
