# Supplementary material for: Genome-wide analysis of mono-, di- and trimethylation of histone H3 lysine 4 in Arabidopsis thaliana
Source: Genome Biol. 2009 Jun 9;10(6):R62. doi: 10.1186/gb-2009-10-6-r62 (PMC2718496; doi:10.1186/gb-2009-10-6-r62)
Supplement: Additional data file 1 — Figure S1: dot blot analysis showing the specificity of antibodies used here. Figure S2: comparison of the H3K4me distribution patterns determined here and those reported in a recent locus-specific study [38]. Figure S3: real-time PCR validation of H3K4me ChIP-chip results. Figure S4: length distribution of H3K27me3 target genes. Table S1: real-time PCR primer sequences. [file gb-2009-10-6-r62-S1.pdf]

SUPPLEMENTAL FIGURE LEGENDS

Figure S1. Dot blot analysis showing the specificity of antibodies used in this study. Peptide concentration is shown on the left.

Figure S2. The H3K4me distribution patterns determined here are highly consistent with results at three loci reported in a recent locus-specific study of H3K4me2 and H3K4me3 [38]. The *NAP* and *WRKY70* genes were reported to contain H3K4me2 and H3K4me3, the *XTH33* gene was reported to contain H3K4me2 but not H3K4me3.

Figure S3. Real-time PCR validation of H3K4me ChIP-chip results in two regions on chromosome 1. ChIP-chip results are shown on top (filled red boxes represent regions amplified by real-time PCR). Real-time PCR results are shown at the bottom. y-axis: fold of enrichment calculated using regions 4 as internal standard (see *Materials and Methods* for details).

Figure S4. Length distribution of H3K27me3 target genes. Only “isolated genes” are included in this analysis to avoid ambiguity. a-axis: gene length (in 200-bp bins). y-axis: percentage of all genes (black) or H3K27me3 target genes (red) with corresponding length.

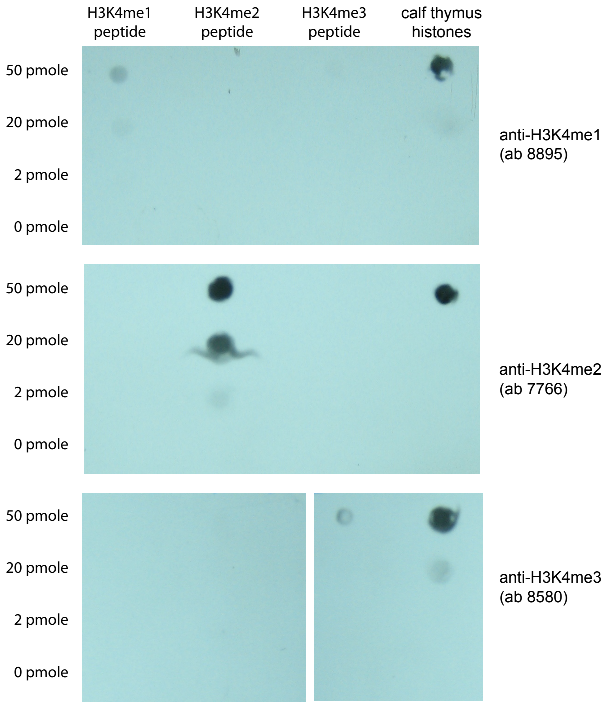
H3K4me1 peptide
H3K4me2 peptide
H3K4me3 peptide
calf thymus histones
50 pmole
20 pmole
2 pmole
0 pmole
anti-H3K4me1 (ab 8895)
anti-H3K4me2 (ab 7766)
anti-H3K4me3 (ab 8580)

Figure S1

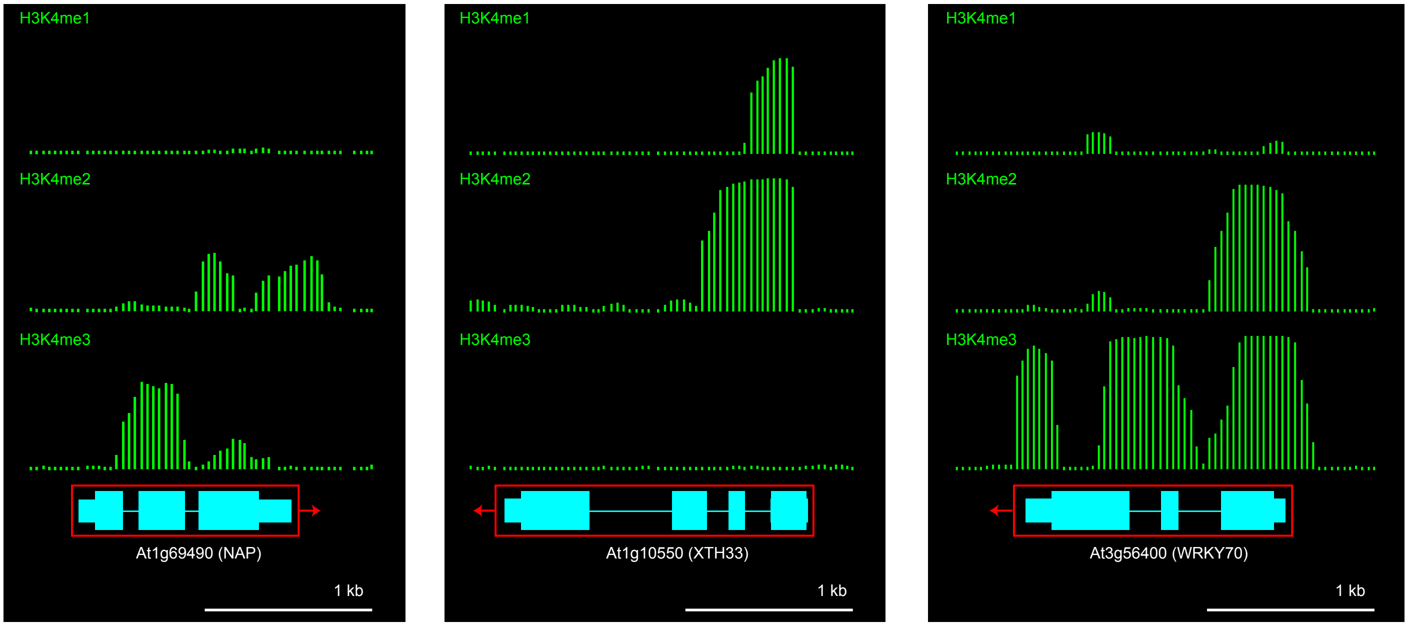
H3K4me1
H3K4me2
H3K4me3
At1g69490 (NAP)
1 kb
H3K4me1
H3K4me2
H3K4me3
At1g10550 (XTH33)
1 kb
H3K4me1
H3K4me2
H3K4me3
At3g56400 (WRKY70)
1 kb

Figure S2

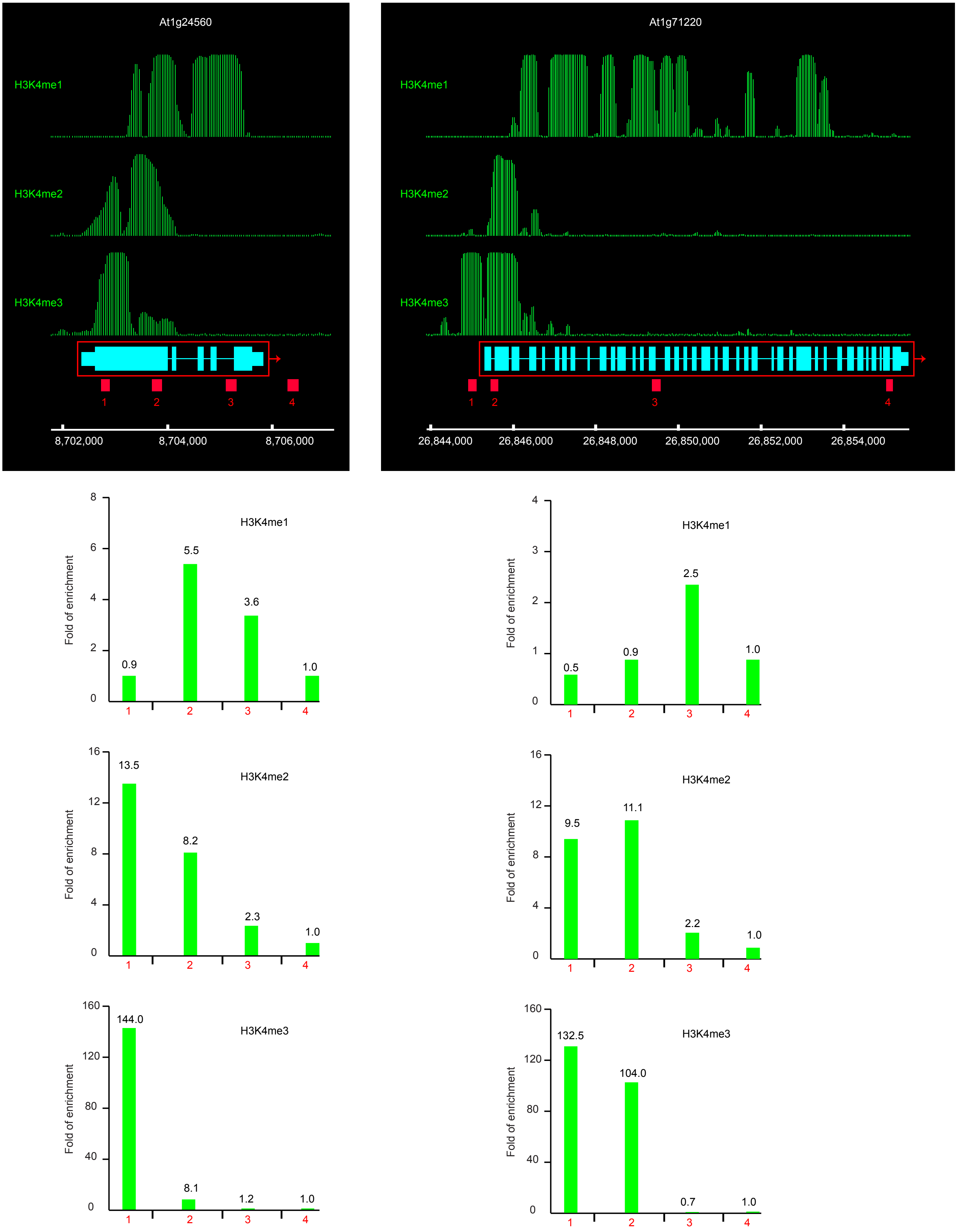
At1g24560
H3K4me1
H3K4me2
H3K4me3
1
2
3
4
8,702,000
8,704,000
8,706,000
At1g71220
H3K4me1
H3K4me2
H3K4me3
1
2
3
4
26,844,000
26,846,000
26,848,000
26,850,000
26,852,000
26,854,000
Fold of enrichment
H3K4me1
0.9
5.5
3.6
1.0
0.5
0.9
2.5
1.0
H3K4me2
13.5
8.2
2.3
1.0
9.5
11.1
2.2
1.0
H3K4me3
144.0
8.1
1.2
1.0
132.5
104.0
0.7
1.0

Figure S3

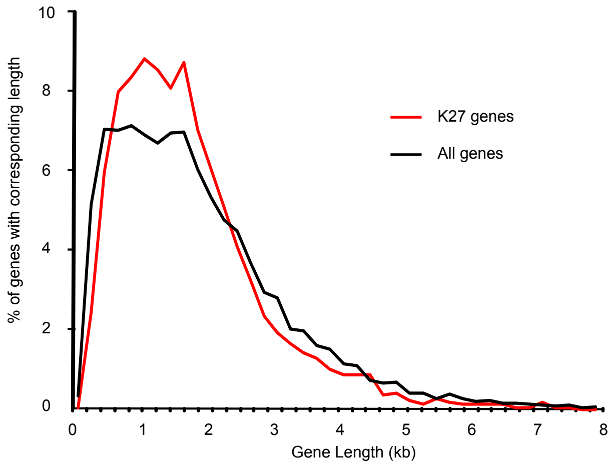

10
8
6
4
2
0
% of genes with corresponding length
K27 genes
All genes
0
1
2
3
4
5
6
7
8
Gene Length (kb)

Figure S4

Table S1. PCR primers used for real-time PCR validation of ChIP-chip results.

Gene	Locus	Forward Primer	Reverse Primer
At1g24560	1	GGAGCTAATAGCGGAGCTTG	TCCTTCAATGCTTCATCACG
At1g24560	2	GCACAGAGTTTGGTGGAAGAG	CACCATCCCTCAAACCATTC
At1g24560	3	GATTGGGAATTGCACTGAGG	ATTGCTTGCTTTGCCTCTTC
At1g24560	4	ACCAAGTCTAGCATGGGAGAGA	TCGAGGGTTTGATTTACATGG
At1g71220	1	TGGCTCTGTTTCACATCTCG	AGATCATGCGAAGGAATGCT
At1g71220	2	TGAGGCTGGGTAATGCTTCT	AATCGGAATCATCTCCATCG
At1g71220	3	TGAAGCAAGCTCCATGTTTG	GTGAACGTCGATGCAGATCA
At1g71220	4	TGTGAATCCTGGTGTGGAAA	TGCAGAAGAACACCGCATTA
